# Clinical-grade mesenchymal stem cells derived from umbilical cord improve septic shock in pigs

**DOI:** 10.1186/s40635-018-0194-1

**Published:** 2018-08-08

**Authors:** Caroline Laroye, Jérémie Lemarié, Amir Boufenzer, Pierre Labroca, Lisiane Cunat, Corentine Alauzet, Frédérique Groubatch, Clémence Cailac, Lucie Jolly, Danièle Bensoussan, Loïc Reppel, Sébastien Gibot

**Affiliations:** 1 CHRU de Nancy, Unité de Thérapie Cellulaire et banque de tissus, 54500 Vandoeuvre-lès-Nancy, France; 2 INSERM, U1116, 54500 Vandoeuvre-lès-Nancy, France; 3 CNRS, UMR 7365, 54500 Vandoeuvre-lès-Nancy, France; 4 Université de Lorraine, 54000 Nancy, France; 5 CHRU de Nancy, Service de Réanimation Médicale, Hôpital Central, 54000 Nancy, France; 6 INOTREM, 54500 Vandoeuvre-lès-Nancy, France; 7 EA 7300 Stress Immunité Pathogènes, 54500 Vandoeuvre-lès-Nancy, France; 8 Ecole de chirurgie, 54500 Vandoeuvre-lès-Nancy, France; 9 CHRU de Nancy, laboratoire anatomie et cytologie pathologiques, 54000 Nancy, France

**Keywords:** Septic shock, Mesenchymal stem cells, Umbilical cord, Clinical-grade

## Abstract

**Background:**

Septic shock is the leading cause of death in intensive care units. The pathophysiological complexity of this syndrome contributes to an absence of specific treatment. Several preclinical studies in murine models of septic shock have shown improvements to organ injury and survival after administration of mesenchymal stem cells (MSCs). To better mimic a clinical approach in humans, we investigated the impact of randomized controlled double-blind administration of clinical-grade umbilical cord-derived MSCs to a relevant pig model of septic shock.

**Methods:**

Septic shock was induced by fecal peritonitis in 12 male domestic pigs. Animals were resuscitated by an experienced intensivist including fluid administration and vasopressors. Four hours after the induction of peritonitis, pigs were randomized to receive intravenous injection of thawed umbilical cord-derived MSCs (UCMSC) (1 × 10^6^ UCMSCs/kg diluted in 75 mL hydroxyethyl starch (HES), (*n* = 6) or placebo (HES alone, *n* = 6). Researchers were double-blinded to the treatment administered. Hemodynamic parameters were continuously recorded. Gas exchange, acid-base status, organ function, and plasma cytokine concentrations were assessed at regular intervals until 24 h after the onset of peritonitis when animals were sacrificed under anesthesia.

**Results:**

Peritonitis induced profound hypotension, hyperlactatemia, and multiple organ failure. These disorders were significantly attenuated when animals were treated with UCMSCs. In particular, cardiovascular failure was attenuated, as attested by a better mean arterial pressure and reduced lactatemia, despite lower norepinephrine requirements. As such, UCMSCs improved survival in this very severe model (60% survival vs. 0% at 24 h).

**Conclusion:**

UCMSCs administration is beneficial in this pig model of polymicrobial septic shock.

## Background

Sepsis, defined as life-threatening organ dysfunction caused by a deregulated host response to infection, is a leading cause of admission to intensive care units and is associated with high mortality rates [1, 2]. Unfortunately, due to the extremely complex physiopathology of sepsis and septic shock, there is still no specific treatment for this syndrome. Indeed, in the initial stages, simultaneous production of pro-inflammatory and anti-inflammatory mediators is observed [3, 4], and thus, it is now accepted that sepsis is not a succession of hyper- and hypo-inflammatory states, but that the two are intertwined. This new paradigm may explain the failure of many therapies targeting this deregulated host response [5].

Among emerging treatments, mesenchymal stem cells (MSCs) appear as promising candidates to treat sepsis and septic shock. MSCs are characterized as non-hematopoietic stem cells, capable of differentiating into chondrocytes, adipocytes, and osteocytes. Their extensive immunomodulatory properties mean they can modulate inflammatory and immune disorders [6–8]. Mei et al. [9] were the first to suggest that MSCs improve survival and organ failure in a mouse model of endotoxemia. The mechanism responsible for the protective action of MSC during septic shock is unclear but appears to be multifactorial. Several studies indicated that MSCs can increase bacterial clearance [10] and modulate cytokine production by decreasing pro-inflammatory mediators and increasing anti-inflammatory cytokines [11–16]. Finally, through their immunomodulatory capacities and antibacterial properties, they can improve renal, pulmonary, liver, cardiac, and muscular function, as well as coagulopathy associated with septic shock [11, 17–21].

However, these studies mostly used MSCs derived from adult tissues (bone marrow and adipose tissue). Adult MSCs present many drawbacks with regard to their potential for clinical applications; the number of adult MSC donors is limited, and adult MSC remain difficult to produce [22]. In contrast, fetal tissues, and particularly umbilical cord (UC), appear to have greater potential as sources of MSCs. Umbilical cord can be donated without risk and is abundantly available. Moreover, MSCs are present in large numbers in the UC, and they can be rapidly and quite easily expanded [23].

In this study, we performed a randomized controlled double-blind investigation to examine the effect of the administration of thawed clinical-grade UC-derived MSCs in a relevant pig model of septic shock resuscitated by an experienced intensivist. We hypothesized that clinical-grade UCMSC would improve hemodynamic parameters and septic shock-induced organ injury.

## Methods

### UCMSC production

Umbilical cords were collected at Nancy Maternity Hospital from new mothers who had signed an informed consent form in compliance with French national legislation regarding human sample collection, manipulation, and personal data protection. The collection protocol was approved by the local ethics committee and the French ministry for research (No. DC-2014-2114). All UCMSCs were produced at clinical-grade in α-MEM culture medium (Macopharma, Mouvaux, France) enriched with 5% platelet lysate (Macopharma, Mouvaux, France) and applying good manufacturing practices. Briefly, UCs were immersed in an antibiotic-antifungal solution composed of gentamicin, amoxicillin, vancomycin, and amphotericin B for 1 h at room temperature. The cord was then cut into thin pieces which were placed in complete medium. The culture was carried out at 37 °C and in hypoxic conditions (5% of O_2_ and 5% of CO_2_). UCMSCs were cultured until passage three and then frozen and stored in vapor phase nitrogen. After thawing, UCMSCs were washed once in hydroxyethyl starch to remove the cryoprotectant and used within 2 h.

### Characterization of UCMSCs

Once 80% confluence was reached, UCMSCs were washed with HBSS and detached by trypsinization. To examine expression levels of surface markers, 1 × 10^6^ UCMSCs were labeled with anti-CD90, CD73, CD44, CD105, CD34, CD45, CD11b, CD19, and HLA-DR mAbs (Stemflow hMSC Analysis kit, Becton Dickinson, Franklin Lakes, USA).

Osteogenic and adipogenic differentiation was also performed to characterize MSCs.

Osteogenic differentiation was induced by seeding UCMSCs at a density of 3100 cells/cm^2^ and maintaining them in culture for 28 days in an osteogenic induction medium (Lonza, Walkersville, USA). After 28 days, samples were fixed in 4% paraformaldehyde and then included in paraffin before staining with alizarin red. To induce adipocyte differentiation, 21,000 UCMSCs/cm^2^ were seeded on 24-well plates. When 100% confluence was reached, 3 induction/maintenance cycles were performed. One induction/maintenance cycle consisted in 3-day culture in induction medium (Lonza, Walkersville, USA), followed by 1 to 3 days of culture in maintenance medium (Lonza, Walkersville, USA). After 3 cycles of induction/maintenance, the cells were cultured for 7 days in complete maintenance medium (Lonza, Walkersville, USA) before staining with oil red.

### Animal preparation

Experiments were performed in line with the National Institute of Health guidelines on the Use of Laboratory Animals and were approved by the University Animal Care Committee (Comité d’Éthique Lorrain en Matière d’Expérimentation Animale (CELMEA - CE2A-66), authorization number: APAFIS5674-201606141602993).

Six-month-old male domestic pigs (40–60 kg) were purchased from Elevage Ferry (Vosges, France). Before surgery, animals were fasted overnight with free access to water. Pre-anesthesia was performed by intramuscular administration of ketamine (10 mg/kg) and midazolam (0.1 mg/kg). Anesthesia was induced and maintained until the end of the protocol with intravenous propofol (8 mg/kg/h), sufentanil (5 μg/h), and cisatracurium (20 mg/h). Animals were mechanically ventilated (tidal volume 8 ml/kg, PEEP 5 cm H_2_O, FiO_2_ 0.30, respiratory rate 14–16 breaths/min adjusted to maintain normocapnia). The right jugular vein was exposed, and a triple-lumen line was inserted. A Swan-Ganz catheter was positioned allowing cardiac output, S_v_O_2_, and right atrial and pulmonary arterial pressure to be continuously recorded. A catheter was inserted in the right carotid artery to continuously measure arterial pressure. A catheter inserted into the bladder was used to collect urine.

After instrumentation, a midline laparotomy was performed to collect feces from the left colon; 3 g/kg was suspended in 200 mL 0.9% NaCl and 50 mL glucose 5% and incubated at 37 °C for 2 h. After surgery, a tube was left in place to induce peritonitis.

Animals were allowed to recover for 2 h after surgery before performing baseline measurements (defined as “H0”). Normal saline was continuously administered (10 mL/kg/h) throughout the study.

#### Experimental protocol

After collecting baseline data (H0), peritonitis was induced by administering autologous feces through the abdominal tube which was subsequently clamped. After 4 h (H4), animals were randomly assigned to groups to receive 1 × 10^6^ UCMSCs/kg diluted in 75 mL hydroxyethyl starch (HES) (*n* = 6) or HES alone (*n* = 6) through the intravenous catheter. Cells or starch were administered over 10 min.

The intensivist in charge of the animal was *blinded* to the treatment administered, which was prepared by an independent investigator.

Animal care was provided by an experienced intensive care physician who adhered strictly to the following guidelines throughout the study period:

i) Hemodynamic targets. The main objective was to maintain mean arterial pressure (MAP) above 85 mmHg. To achieve this goal, 0.9% NaCl was administered (up to 20 mL/kg) provided that central venous pressure (CVP) and pulmonary artery occlusion pressure (PAOP) were < 18 mmHg. When the maximum volume was reached, continuous infusion of norepinephrine was started up to 10 μg/kg/min.
ii) Respiratory targets. The main objective was to maintain a PaO_2_/FiO_2_ ratio > 300 and an arterial PaCO_2_ at 35–45 mmHg. Ventilator settings could thus be modified by increasing inspiratory/expiratory ratio close to 1:1, PEEP up to 15 cm H_2_O, and respiratory rate up to 30 breaths/min.
iii) Body temperature was kept constant (± 1 °C) using heating pads or cooling.
iv) Intravenous glucose infusion was administered when necessary to maintain glycemia at 5–7 mmol/L.

Animals were sacrificed under deep anesthesia by KCl infusion 24 h after the induction of peritonitis or if MAP remained < 50 mmHg during more than 30 min despite administration of the maximum dose of norepinephrine.

#### Measurements

Hemodynamic parameters were continuously monitored including MAP, mean pulmonary artery pressure (MPAP), right atrial pressure (RAP), cardiac output (CO), cardiac index (CI), and S_v_O_2_.

Blood was sequentially drawn for the determination of (i) blood gases, (ii) arterial lactate, (iii) plasma concentration of urea, creatinine, albumin (VetTest GHP, Idexx, Saint-Denis, France) (iv) blood cell count, and (v) TNF-α, and IL-6 (ELISA, RnD Systems, Minneapolis, USA). At the end of the experiment, bacterial counts were performed on blood samples, and lung and kidney biopsies were taken for histological analyses. Histology scores were determined by an experienced pathologist who was blinded to the treatment applied.

#### Statistics

After testing for their normal distribution (Kolmogorov-Smirnov test), data were presented as mean ± SEM. Between-group differences were analyzed for statistical significance by two-way ANOVA for repeated measures, using the Bonferroni correction or Student’s *t* test when appropriate. Statistical analyses were performed using GraphPad Prism software.

## Results

### Characteristics of UCMSCs

**Fig. 1:**
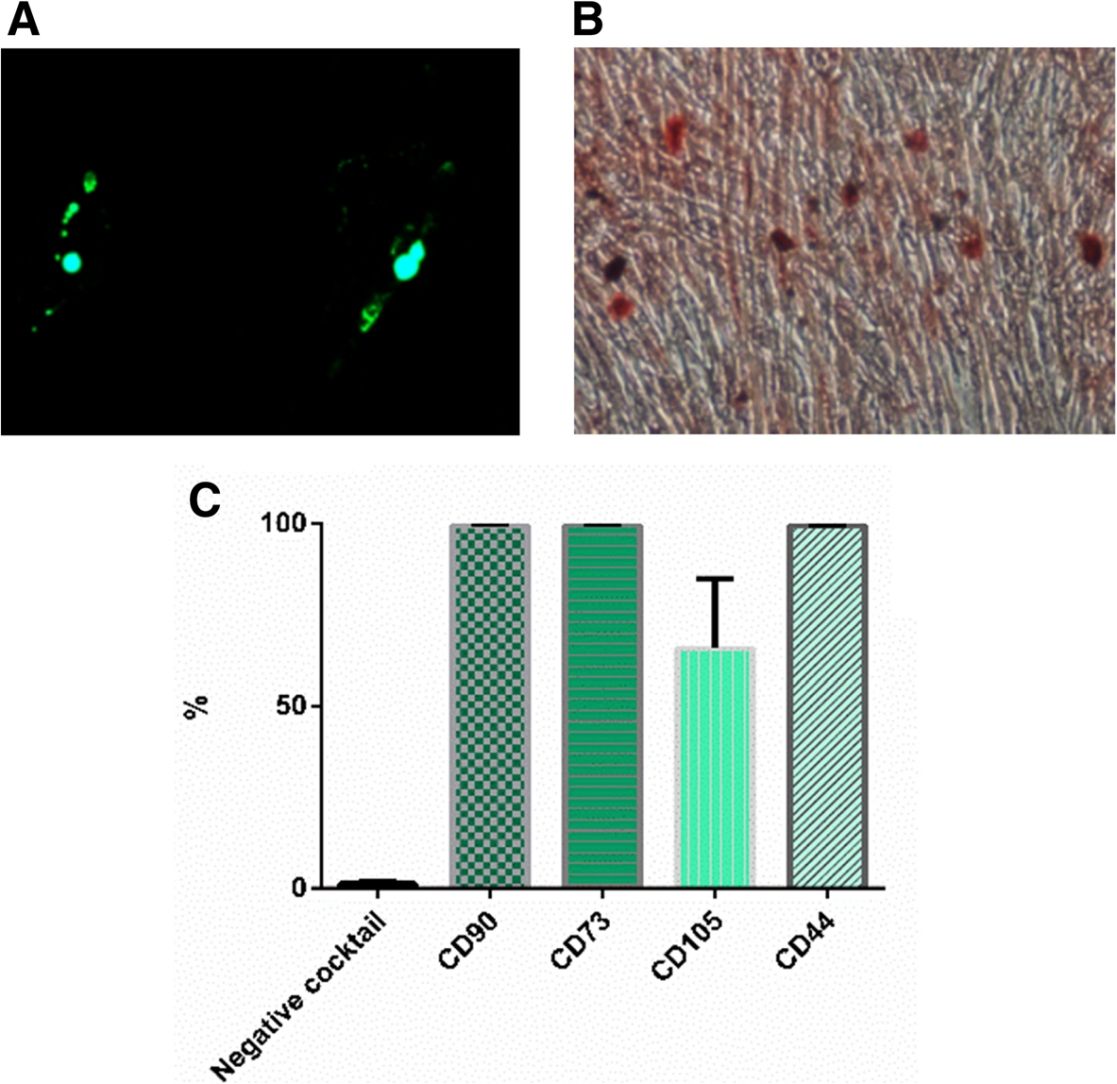
Characterization of UCMSCs. Mesodermic differentiation of UCMSCs. Clinical-grade UCMSCs differentiated into adipocytes (**a**) and osteocytes (**b**). Representative images are shown at × 10 magnification. Immunophenotypic analysis of UCMSCs by flow cytometry (**c**). UCMSCs presented the typical immunophenotype of MSCs. Negative cocktail includes CD34, CD45, CD11b, CD19, and HLA-DR markers. Results are shown as percentages of positive cells and are expressed as mean ± SEM. (*n* = 3)

According to the standards described by the International Society for Cellular Therapy [24], the immunophenotype for UCMSCs is CD14^neg^-CD34^neg^-HLA-DR^neg^-CD11b^neg^-CD19^neg^-CD73^+^-CD90^+^-CD105^+^-CD44^+^. These cells can also be induced to differentiate into osteocytes and adipocytes (Fig. 1).

### UCMSCs attenuate hemodynamic failure associated with septic shock

**Fig. 2:**
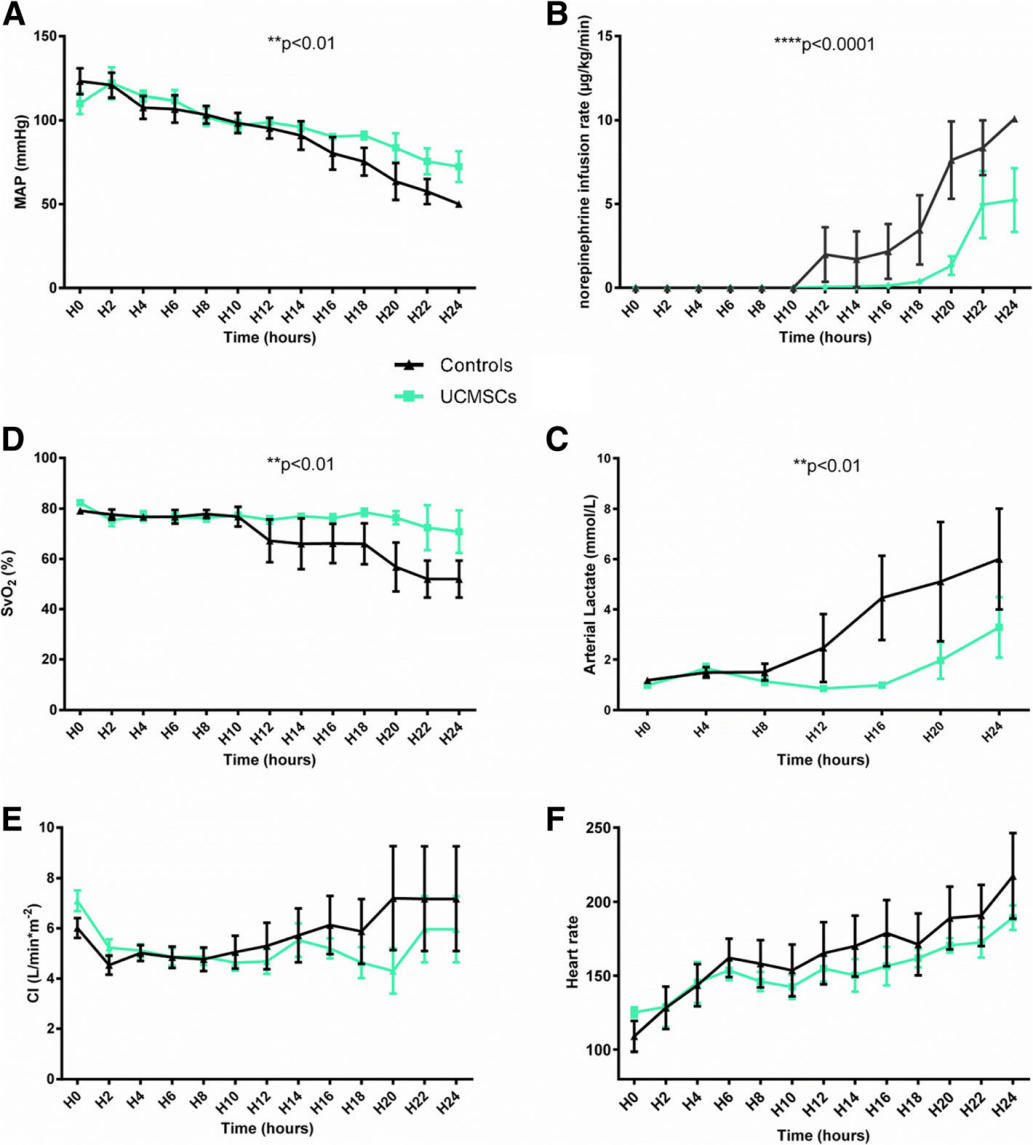
UCMSCs protect from sepsis-induced hypotension and tissue hypoxia. Evolution of (**a**) mean arterial pressure (MAP), (**b**) norepinephrine requirements, (**c**) SvO2, (**d**) arterial lactate concentration, (**e**) cardiac index (CI), and (**f**) heart rate over the 24-h study period. MAP and SvO_2_ were higher in UCMSC-treated animals than controls; whereas, norepinephrine dose and lactate levels were lower. (*n* = 6 per group).^*^p < 0.05; ^**^p < 0.01;^***^p < 0.001; ^****^p < 0.0001 versus control group

Peritonitis induced a rapid drop in MAP (Fig. 2) despite volume resuscitation (8.16 mL/kg/h for controls vs. 7.48 mL/kg/h for UCMSC group, *p* = 0.667). Therefore, to maintain MAP > 85 mmHg, norepinephrine was started by H12 in 4/6 and 1/6 control and UCMSC animals, respectively. The norepinephrine infusion rate required to maintain blood pressure was significantly lower in the UCMSC-treated animals than in controls (*p* < 0.0001) (Fig. 2).

Although no differences were observed between groups for CO and HR, SvO_2_ was significantly improved and lactate concentrations significantly reduced in the group receiving UCMSCs (*p* < 0.01), suggesting lower tissue hypoxia in this group (Fig. 2).

### UCMSCs ameliorate organ failure

**Fig. 3:**
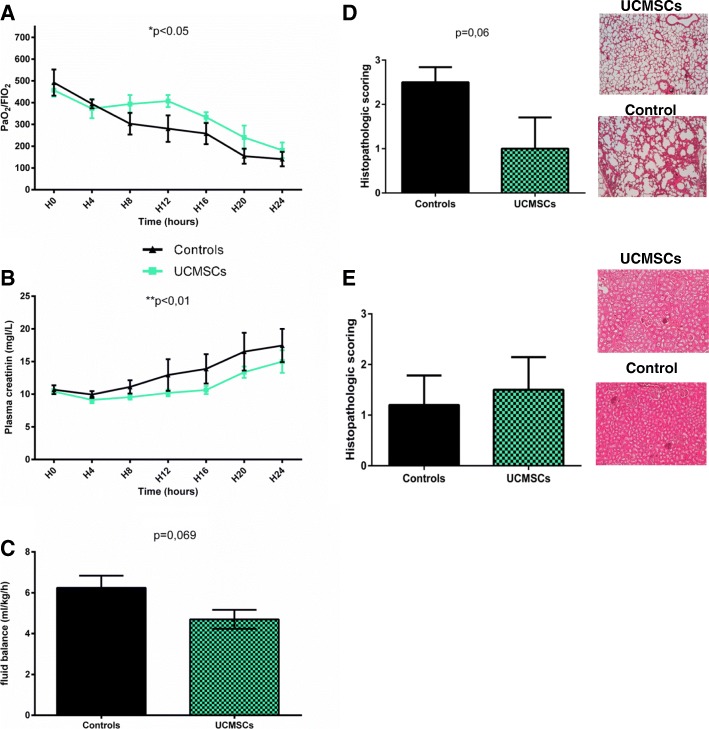
UCMSCs attenuate sepsis-induced organ failure. Lung function was monitored by assessing the PaO_2_/FIO_2_ ratio (**a**) and based on histology (**d**). Renal function was assessed by measuring plasma creatinine concentration (**b**), fluid balance (**c**), and by histological observation (**e**). For histological scoring, a score of 0 corresponds to an absence of anomaly, 1 reflects discrete anomalies, 2 corresponds to moderate anomalies, and 3 indicates severe anomalies. Histological images (hematoxylin-eosin staining, × 40 magnification) are representative of the respective conditions. ^*^p < 0.05; ^**^p < 0.01;^***^p < 0.001; ^****^p < 0.0001 versus control group

Sepsis induced progressive hypoxemia reflected by a decrease in the PaO_2_/FiO_2_ ratio (Fig. 3a). This phenomenon was significantly attenuated in the UCMSC group (*p* < 0.05). Histological observations supported this finding, as reduced inflammatory infiltration and interstitial edema was recorded in treated pigs.

UCMSC administration appears to have a moderate effect on kidney failure. Despite a trend for improved diuresis, and a significantly smaller increase in plasma creatinine (*p* < 0.01) (Fig. 3b), histological examinations revealed no differences between the two groups.

### UCMSCs show no antimicrobial effect

**Fig. 4:**
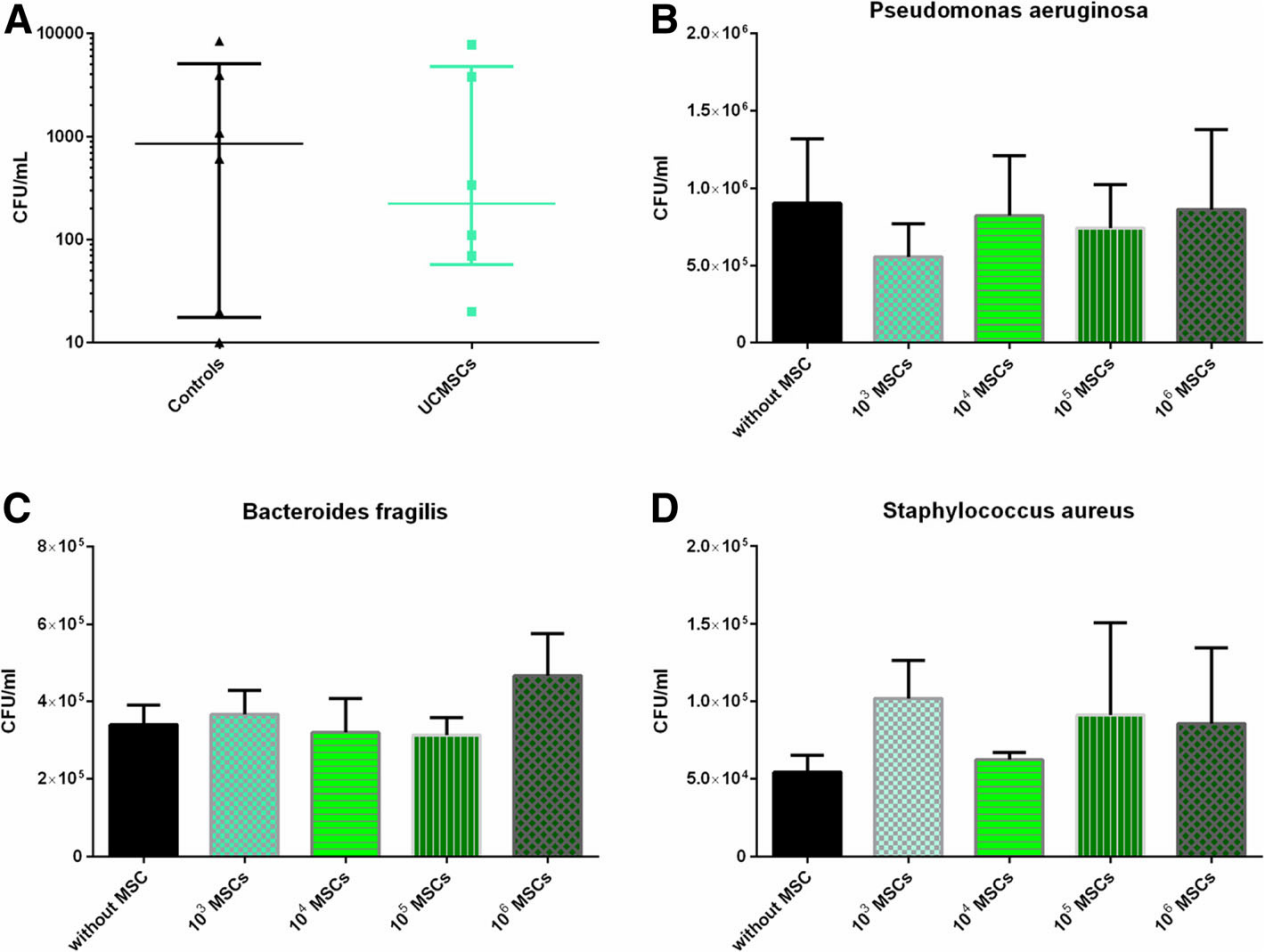
UCMSCs have no antibacterial effect. Quantitative blood cultures at the time of death (**a**). The graph represents the medians and IQR of bacterial CFU (*n* = 6 per group). UCMSCs were co-cultured at different concentrations with 10^4^ to 10^5^ CFU of three species of bacteria commonly found in intestinal microbiota: *Pseudomonas aeruginosa* (**b**), *Bacteroides fragilis* (**c**), and *Staphylococcus aureus* (**d**) (*n* = 3 per group). CFU counts were determined by serial dilution and agar plating after 3 h of co-culture. Differences between groups were not statistically significant

All septic animals were found to be bacteremic. No significant differences were observed between groups. Median bacterial colony-forming unit (CFU) counts were 855 CFU/ml of blood for controls and 225 CFU/ml of blood for UCMSC-treated animals (Fig. 4a).

To determine if this result was due to inadequate numbers of infiltrated UCMSC, we tested the antibacterial properties of UCMSC at different concentrations in vitro (Fig. 4b–d). No antimicrobial effect of UCMSCs was observed on the three bacterial species tested.

### UCMSCs have no effect on cytokine production

**Fig. 5:**
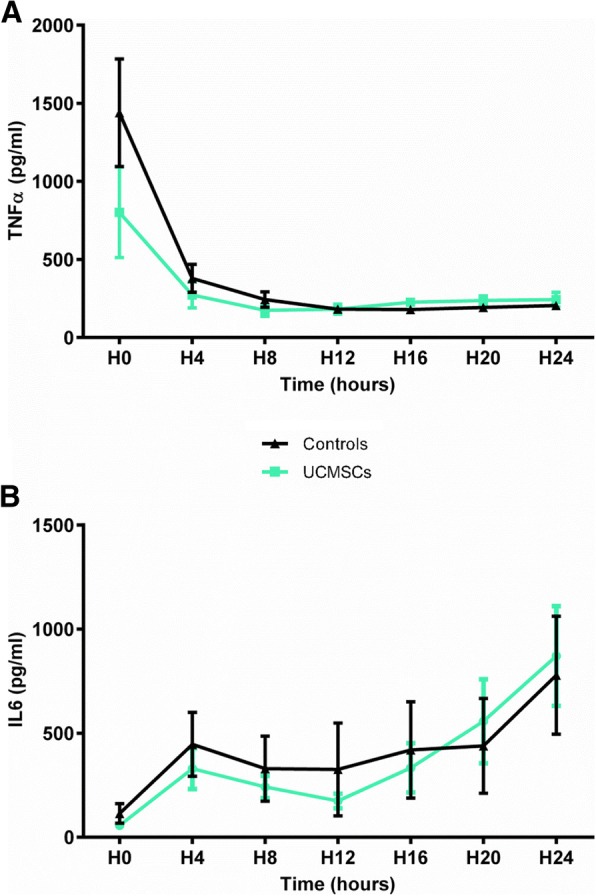
UCMSCs have no effect on systemic cytokine concentrations. Plasma concentrations of TNFα (**a**) and IL-6 (**b**) were similar between groups (*n* = 6 per group)

To determine the impact of UCMSCs on inflammation, we measured plasma concentrations of IL-6 and TNFα. TNFα levels, high at the beginning of study due to the surgical procedure, decreased during the first 4 h of peritonitis and then stabilized until the end of the protocol. The TNFα profile was similar for both groups (Fig. 5a). IL-6 concentrations progressively increased throughout the study period, both in the treated and the control groups (Fig. 5b). UCMSCs had no effect on IL-6 or TNFα.

### UMSCs do not alter blood immune profile

After induction of peritonitis, slight leukopenia with a drop in granulocyte numbers was observed in both groups; differences were not significant. Thrombocytopenia was also observed, but was not influenced by UCMSC treatment (Table 1).

**Table 1:** Effects of UCMSCs on leukocytes and platelets count

	H0	H8	H16	H24
Leukocytes cells/mm^3^, control	18,000 (1480)	11,400 (1067)	15,100 (3342)	13,333 (3580)
UCMSCs	22,900 (3342)	11,400 (1106)	12,533 (1233)	15,600 (3323)
Lymphocytes cells/mm^3^, control	5872 (407)	5227 (729)	6318 (1141)	4775 (316)
UCMSCs	6401 (713)	4502 (587)	4698 (608)	6577 (922)
Monocytes cells/mm^3^, control	1639 (573)	1657 (283)	2563 (926)	3024 (1354)
UCMSCs	2954 (806)	1489 (246)	1518 (998)	2403 (520)
Granulocytes cells/mm^3^, control	10,800 (1973)	5749 (1059)	8517 (3162)	5532 (1925)
UCMSCs	8967 (1796)	5750 (741)	4199 (833)	6618 (2217)
Platelets / mm^3^, control	391,167 (36238)	356,667 (24503)	273,667 (42942)	195,750 (37666)
UCMSCs	444,400 (42887)	369,500 (45603)	264,000 (25923)	226,400 (38203)

### UCMSCs improve survival

**Fig. 6:**
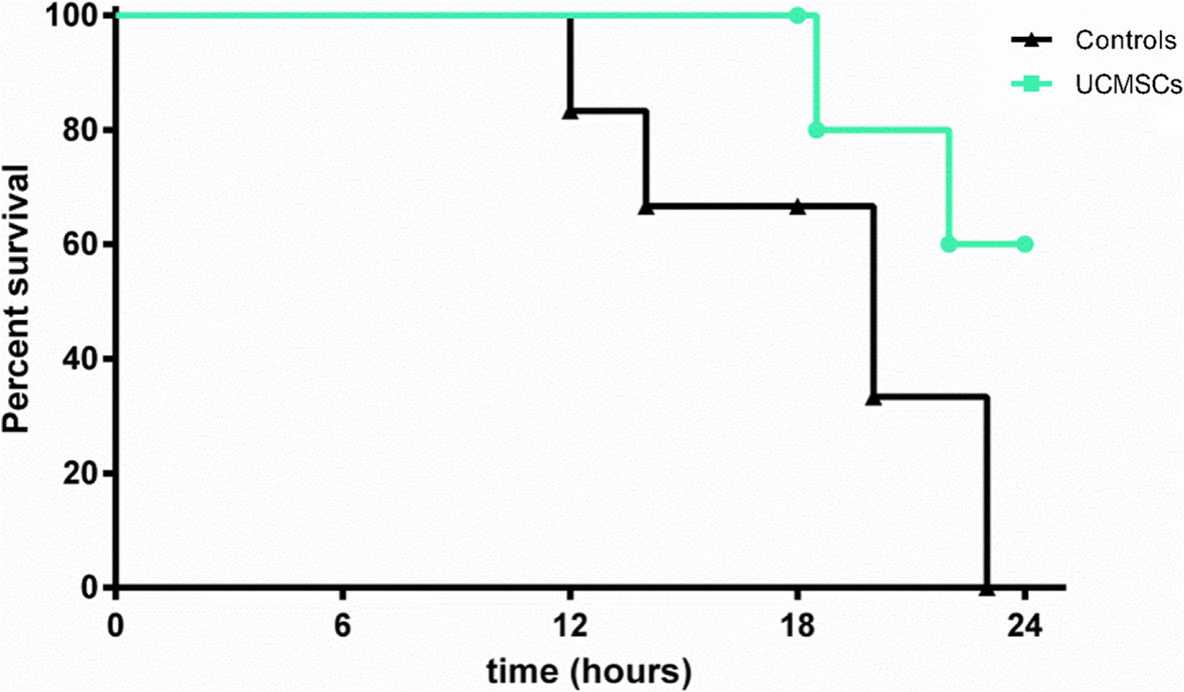
UCMSCs improve survival. Survival percentages of untreated and treated pigs after induction of peritonitis are presented as a Kaplan-Meier survival curve (Log-Rank test, *p* = 0.08)

Twenty-four hours after induction of peritonitis, all control animals had died; whereas in the UCMSC group, 60% of pigs were still alive at the end of the protocol (Log-rank test, *p* = 0.08) (Fig. 6).

## Discussion

Many preclinical studies have reported a beneficial effect of administering MSC during septic shock. However, these murine studies did not reflect human clinical practice and generally used MSCs derived from adult tissues (bone marrow and adipose tissue). Here, we tried to avoid these pitfalls and to better mimic a clinical approach in humans. We thus applied a randomized controlled double-blind protocol to investigate the effect of administering thawed clinical-grade UC-derived MSCs in a relevant pig model of septic shock resuscitated by an experienced intensivist. UCMSCs improved survival, hemodynamic parameters (reflected by a reduced requirement for norepinephrine infusion), and organ injury. All these improvements are highly relevant from a clinical perspective.

According to previously published preclinical studies, the protective effects of MSCs are closely related to their immunomodulatory capacities. Reports indicate reduced production of pro-inflammatory cytokines and increased levels of anti-inflammatory cytokines, which may modulate the innate and adaptive immune responses during septic shock [13, 16, 25–27]. Another reason advanced to explain the beneficial effect of MSCs is their antibacterial properties. Several authors demonstrated that MSC administration improves bacterial clearance by triggering increased neutrophil phagocytosis and production of antibacterial peptides such as LL-37 and hepcidin [10, 28–30]. Our results contrast with these previous findings as we did not observe any effect of MSCs on cytokine production or bacterial clearance. This discrepancy may be explained by differences between the animal models used and MSC doses. Indeed, most murine studies used very large MSC doses. For example, Song et al. [25] indicated that administration of 40.10^6^ MSCs/kg (40-fold higher than in our study) decreased plasma levels of IL-6 and TNFα, increased IL-10 and improved bacterial clearance during CLP. Similarly, studies demonstrating antibacterial properties of MSC in vitro used an excess of cells relative to bacterial CFU [28, 31]. Our data indicate no antibacterial or anti-inflammatory action for UCMSC in vivo or in vitro when used at a clinically relevant dose.

However, it is surprising that despite the lack of effect on inflammation and bacterial clearance, treated pigs exhibited lower organ failure and improved survival. Indeed, the most impressive effect was observed on MAP which was better preserved with reduced requirement for vasopressors. We can speculate that this effect on vascular tone stems from a role of UCMSCs in preventing sepsis-induced endothelial dysfunction. A protective effect of MSCs on endothelial cells has recently been suggested. Yang et al. [32] showed that infiltration of MSCs protected lung endothelium from apoptosis and maintained adherens junctions in a murine model of acute lung injury. This improvement to lung permeability correlated with an increase in VEGF levels. Using the same animal model, Xiang et al. [33] observed up-regulation of VE-cadherin and catenin in MSC-treated mice. According to Hu et al. [34], this up-regulation tended to correlate with an increase in hepatocyte growth factor. The protective effect of MSCs could also be the result of mitochondrial transfer. Indeed, MSC can transfer mitochondria to damaged cells through intracellular nanotubes, gap junctions, and cell fusion or carried in microvesicles [35]. This mechanism appears to be related to the improvement to organ failure in various diseases. Indeed, Islam et al. demonstrated that MSC mitochondria transfer protects from lung injury [36]. Rocheteau et al. found an amelioration of mitochondria dysfunction in septic myopathy after injection of MSC [21]. Finally, Cselenyák et al. observed intercellular connections which save damaged cardiomyoblasts [37]. As mitochondria dysfunction is associated in sepsis patients with organ injuries and poor outcome, the MSC mitochondria transfer should be explored to explain their protective effect.

We believe that our study is particularly important in highlighting the action of MSCs during sepsis and septic shock for several reasons. First, we used a pig model of resuscitated septic shock. The use of this animal model better mimics the clinical situation than murine models both in terms of weights (40–60 kg) and cardiovascular physiology [38]. Second, we performed this study in a double-blind way to avoid bias management, and an experienced intensivist took care of the resuscitation during the whole study. Third, UCMSCs were produced in clinical grade and were used immediately after thawing, reflecting the clinical setting. This point is very important as the effect of cryopreservation on MSCs is unclear. Moll et al. demonstrated that freeze-thawed MSCs decreased response to pro-inflammatory stimuli and lowered their ability to secrete anti-inflammatory cytokines as compared to fresh MSCs [39]. Likewise, François et al. found that freeze-thawed MSCs are refractory to interferon IFNγ-induced up-regulation of indoleamine 2, 3-dioxygenase [40]. However, these authors also indicated that the immunosuppressive activity of MSCs could be restored after a 24-h culture. In contrast, Luetzkendorf et al. [41] showed that if MSCs were used immediately after thawing, they decreased the proliferation of PBMCs stimulated with phytohemagglutinin to a similar extent to fresh MSCs. Finally, Barcia et al. [42] demonstrated in a murine model of arthritis that fresh and frozen MSCs had an identical ability to decrease inflammation. Our results indicate that UCMSCs produced according to good manufacturing practice conditions, cryopreserved and administered intravenously after thawing, could protect against organ failure during septic shock.

However, this study presents some limitations. First, no antibiotics were administered nor lavage of the peritoneal cavity performed. These were deliberate choices as we wished to study a very severe model with 100% mortality, to allow us to determine the potential effects of UCMSCs in the most severe septic shock patients. Second, unlike most septic shock patients, our pigs had no comorbidities. Finally, UCs were not selected based on obstetric factors, as recently reported by Avercenc-Léger et al. [43]. Indeed, birth weight, the number of weeks’ amenorrhea, placental weight, normal pregnancy, and absence of preeclampsia have all been identified as critical factors for cell expansion. Although the optimal selection criteria for UC used for MSC production for inflammation disease treatment are not yet defined, it could be interesting to select UC according to these obstetric factors as part of standardization of production procedures.

## Conclusion

The results presented here show that the administration of thawed clinical-grade UC-derived MSCs significantly improved hemodynamic parameters and organ injury in a relevant pig model of septic shock and improved survival. This first study conducted in large animals indicates that UCMSCs are a promising treatment option for septic shock.

## Abbreviations

CARS: Compensatory anti-inflammatory response
CFU: Colony-forming units
CO: Cardiac output
CVP: Central venous pressure
GvHD: Graft-versus-host disease
HES: Hydroxyethyl starch
IC: Cardiac index
MAP: Mean arterial pressure
MARS: Mixed antagonist response syndrome
MPAP: Mean pulmonary artery pressure
MSC: Mesenchymal stem cells
PAOP: Artery occlusion pressure
RAP: Right atrial pressure
SIRS: Systemic inflammatory response syndrome
UC: Umbilical cord
UCMSC: Umbilical cord-derived mesenchymal stem cells
α-MEM: Minimum essential medium alpha
